# Choline chloride/urea as a green and efficient deep eutectic solvent in three-component and four-component synthesis of novel pyrazole and pyrano[2,3-c] pyrazole derivatives with antibacterial and antifungal activity

**DOI:** 10.3389/fchem.2024.1342784

**Published:** 2024-02-16

**Authors:** Israa Habeeb Naser, Hassan Thoulfikar A. Alamir, Ali Hisham Al-Shukarji, Batool Ali Ahmed, Talal Aziz Qassem, Maher Kamal, Tahani M. Almeleebia, Enas R. Alwaily, Eftikhaar Hasan Kadhum, Ahmed Alawadi, Ali Alsalamy

**Affiliations:** ^1^ Medical Laboratories Techniques Department, Al-Mustaqbal University, Hillah, Iraq; ^2^ Faculty of Pharmacy, Department of Pharmaceutics, University of Al-Ameed, Karbala, Iraq; ^3^ Department of Medical Laboratories Technology, Al-Manara College for Medical Sciences, Maysan, Iraq; ^4^ Department of Medical Engineering, Al-Nisour University College, Baghdad, Iraq; ^5^ Department of Medical Laboratory Technics, Al-Noor University College, Nineveh, Iraq; ^6^ Department of Dentistry, Al-Hadi University College, Baghdad, Iraq; ^7^ Department of Clinical Pharmacy, College of Pharmacy, King Khalid University, Abha, Saudi Arabia; ^8^ Microbiology Research Group, College of Pharmacy, Al-Ayen University, Thi-Qar, Iraq; ^9^ National University of Science and Technology, Dhi Qar, Iraq; ^10^ College of Technical Engineering, The Islamic University, Najaf, Iraq; ^11^ College of Technical Engineering, The Islamic University of Al Diwaniyah, Al Diwaniyah, Iraq; ^12^ College of Technical Engineering, The Islamic University of Babylon, Babylon, Iraq; ^13^ College of Technical Engineering, Imam Ja’afar Al‐Sadiq University, Al‐Muthanna, Iraq

**Keywords:** choline chloride/urea, deep eutectic solvent, pyrazole, pyrano[2,3-c]pyrazole, biological evaluation, antimicrobial agent

## Abstract

In this study, choline chloride/urea was used as a green deep eutectic solvent in the three-component reaction of hydrazine/phenylhydrazine, malononitrile, and aromatic aldehydes for synthesizing pyrazole derivatives, and in the four-component reaction of methyl/ethyl acetoacetate, hydrazine/phenylhydrazine, malononitrile, and aromatic aldehydes for synthesizing pyrano[2,3-c]pyrazole derivatives. Elemental analysis, ^1^H, and ^13^C NMR spectroscopy were used to confirm the structure of the synthesized pyrazole and pyrano[2,3-c] pyrazole derivatives. The antimicrobial effects of the synthesized pyrazole and pyrano[2,3-c] pyrazole derivatives were investigated. In antimicrobial tests, instructions from clinical and laboratory standards institutes were used. Antimicrobial study was done on pathogenic gram-positive and gram-negative species, and specialized aquatic strains and fungal species. Using choline chloride/urea, novel pyrazole derivatives and pyrano[2,3-c]pyrazole derivatives were synthesized, and other derivatives were synthesized with higher efficiency in less time than some previously reported methods. MIC (minimum inhibitory concentration) and MBC (minimum bactericidal concentration) obtained for derivatives were higher than some antibiotic drugs. Synthesis and reports of new derivatives of pyrazole and pyrano[2,3-c]pyrazole, and investigation and reports of their antimicrobial properties on gram-positive, gram-negative, and specialized aquatic and fungal species are among the novel and important findings of this study.

## 1 Introduction

The use of green processes in synthesizing organic and heterocyclic compounds is noteworthy (Nishanth Rao et al., 2021). Green methods in organic synthesis are essential because they are environmentally friendly (Kumar et al., 2020). Among the green techniques in organic chemistry are the synthesis of organic and heterocyclic compounds during multicomponent reactions, the use of recyclable catalysts, and the use of deep eutectic solvents (Calvo-Flores and Mingorance-Sánchez, 2021; Javahershenas, 2023). Multi-component reactions, in which several reactants lead to the synthesis of the desired product in one pot and one step, in addition to being green, are important in the synthesis of organic and heterocyclic compounds due to their high efficiency, cost-effectiveness, and time-saving characteristics (Bhaskaruni et al., 2020; John et al., 2021). There have been several reports of the synthesis of five-membered heterocycles, such as pyrazoles, or two-ring heterocyclic compounds, such as pyranopyrazoles, using multicomponent reactions (Mamaghani and Hossein Nia, 2021; Becerra et al., 2022; Khazaal and Ibraheam, 2022). Pyrazole is a useful heterocyclic compound due to its agricultural and drug-discovery applications (Kumar et al., 2013). There have been reports of this heterocyclic compound in nature, for example, isolation from fungi such as *Colletotrichum gloeosporioides* (Cui et al., 2023). The biological properties of this heterocyclic compound that can be mentioned are its anxiolytic, antidepressant, anti-tubercular, analgesic properties, and anti-malarial activities, etc. (Ramadan et al., 2021). As there is a pyrazole structure in the structure of 4-[5-(4-methylphenyl)-3-(trifluoromethyl)-1h-pyrazol-1-yl]benzene-sulfonamide, which is a drug with the brand name Celecoxib, this heterocyclic compound can be used as a medicinal and bioactive compound (Küçükgüzel and Şenkardeş, 2015). Pyrano[2,3-c]pyrazole derivatives are one of the pyrazole derivatives that contain two heterocyclic compounds of pyrazole and pyran, which are connected by one facet. Derivatives of pyrano[2,3-c] pyrazole can maintain the biological properties of both heterocyclic compounds of pyran and pyrazole. Biological properties, such as cytotoxic, mutagenicity, cancer therapy, and antiviral properties, have been reported for the derivatives of the 6-membered heterocyclic compound of pyran with one oxygen in its structure (Yahiaoui et al., 2022). Biological properties, such as anti-tubercular, anti-malarial, anti-microbial, and anti-cancer properties, have been reported for the derivatives of pyrano[2,3-c]pyrazole (Mokariya et al., 2022; Parikh et al., 2022). In addition to the green method of multicomponent reaction, by using other green techniques such as the use of deep eutectic solvents, pyrazole derivatives and pyrano[2,3-c]pyrazole derivatives can be synthesized, and reports have been made in this field (Nguyen et al., 2021; Sikandar and Zahoor, 2021). As mentioned, the use of the deep eutectic solvent is one of the important and green methods in synthesizing organic and heterocyclic compounds. With this method, there is no need to use dangerous organic solvents or catalysts in the synthesis of organic and heterocyclic compounds (Rushell et al., 2019). A deep eutectic solvent plays the role of solvent and catalyst in the synthesizing of heterocyclic and organic compounds (Fekri et al., 2020). Deep eutectic solvents can be used during multicomponent reactions (Calvo-Flores and Mingorance-Sánchez, 2021). In this regard, we can refer to reports such as the use of glycerol: potassium carbonate in the four-component reaction of malononitrile, carbon disulfide, carbonyl, and methylene compounds, which led to the synthesis of [1,3]dithiine derivatives (Moghaddam-Manesh and Hosseinzadegan, 2021). Another deep eutectic solvent system that has been reported so far for the synthesis of pyrazolopyridines derivatives (Vanegas et al., 2019); tetrahydrobenzo[b]pyran and pyrano[2, 3-d]pyrimidinone (thione) (Biglari et al., 2020); and pyrazolines, pyrimidines, and naphthyridines (Mohamed et al., 2022) is the use of choline chloride/urea. According to reports, using choline chloride/urea reduces the synthesis time of the desired derivatives and has led to higher efficiency to synthesis of new derivatives. Therefore, choline chloride/urea was used as a deep eutectic solvent to synthesize pyrazole derivatives and pyrano[2,3-c]pyrazole derivatives. The synthesis of new derivatives and the experiments of antimicrobial and antifungal properties of the synthesized derivatives were among the other investigations carried out in this study.

## 2 Materials and methods

### 2.1 Equipment and materials

The Brookfield DV-II + Pro EXTRA viscometer was used to measure solvent viscosity, and the G LAB melting point apparatus was used to measure the melting point of solvent and derivatives. The ^1^H, and ^13^C NMR spectra, CHNS/O elemental analyzer, and mass analysis of compounds were prepared using the Varian Inova 500MHz, the EMA 502, and the Agilent technologies 5975C. The Thermo biomate 5 Spectrophotometer was used to prepare the suspension of bacteria. The suspension of bacteria was obtained from the American Type Culture Collection (ATCC). All the reagent materials used for the synthesis of deep eutectic solvent and the synthesis of pyrazole derivatives and pyrano[2,3-c] pyrazole derivatives such as choline chloride, urea, malononitrile, aldehyde derivatives, and hydrazine derivatives were obtained from Merck and Sigma.

### 2.2 Solvent preparation

To prepare the choline chloride/urea deep eutectic solvent, choline chloride and urea were weighed in different ratios, 1:1, 1:2, 1:3, and 1:4, and stirred at 80°C until a colorless homogeneous mixture was observed (Yadav and Pandey, 2014). The prepared mixture was cooled and used for other steps.

### 2.3 Synthesis of pyrazole derivatives

To 1 g choline chloride/urea (1:2) deep eutectic solvent, 1 mmol malononitrile and 1 mmol aldehyde derivatives were added and stirred at 80°C for 5 min. Then 1 mmol hydrazine derivatives was added and stirred at 80°C until the reaction was complete. The completion of the reaction was checked by TLC. Finally, after the completion of the reaction, the temperature was brought to the ambient temperature, and 5 mL of distilled water was added and stirred for 30 min. The sediments, which were the desired product, were separated with filter paper. The desired synthesized product was purified by recrystallization in a mixture of distilled water and ethanol (1:1).

### 2.4 Synthesis pyrano[2,3-c] pyrazole derivatives

To 1 g choline chloride/urea deep eutectic solvent, 1 mmol malononitrile and 1 mmol aldehyde derivatives were added and stirred at 70°C for 5 min. To 1 g choline chloride/urea deep eutectic solvent, 1 mmol ethyl acetoacetate and 1 mmol hydrazine derivatives were added and stirred at 80°C for 5 min. The mixtures were added together and stirred at 80°C until the reaction was complete. The completion of the reaction was checked by TLC. Finally, after the completion of the reaction, the temperature was brought to the ambient temperature, and 5 mL of distilled water was added and stirred for 30 min. The sediments, which were the desired product, were separated with filter paper. The desired synthesized product was purified by recrystallization in a mixture of distilled water and ethanol (1:1).

### 2.5 Antimicrobial and antifungal activity pyrazole derivatives and pyrano[2,3-c]pyrazole derivatives

Based on previous studies reported according to relevant standards, the antimicrobial activity of synthetic derivatives on *Streptomyces fradiae* (10745), *Staphylococcus aureus* (29213), *Streptococcus pyogenes* (19615), *Streptococcus agalactiae* (12386), *Yersinia enterocolitica* (9610), *Shigella dysenteriae* (13313), *Pseudomonas aeruginosa* (15442), *Acinetobacter baumannii* (19606), *Streptococcus iniae* (29178), *Loctococcus garvieae* (43921), *Yersinia ruckeri* (29473), *Edwardsiella tarda* (15947), *Candida albicans* (10231), *Cryptococcus neoformans* (32045), *Fusarium oxysporum* (7601), and *Aspergillus fumigatus Fresenius* (1022) was investigated and evaluated. For this purpose, first, the half-McFarland concentration of the strains and the initial concentrations of 1, 2, 4, 8, 16, 32, 64, 128, 256, 512, 1024, and 2048 μg/mL of the derivatives were prepared separately. The minimum inhibitory concentration (MIC), minimum bactericidal concentration (MBC), minimum fungicidal concentration (MFC), and inhibition zone diameter (IZD) were evaluated (Igei et al., 2016; Suksatan et al., 2022).

## 3 Results and discussion

**SCHEME 1:**
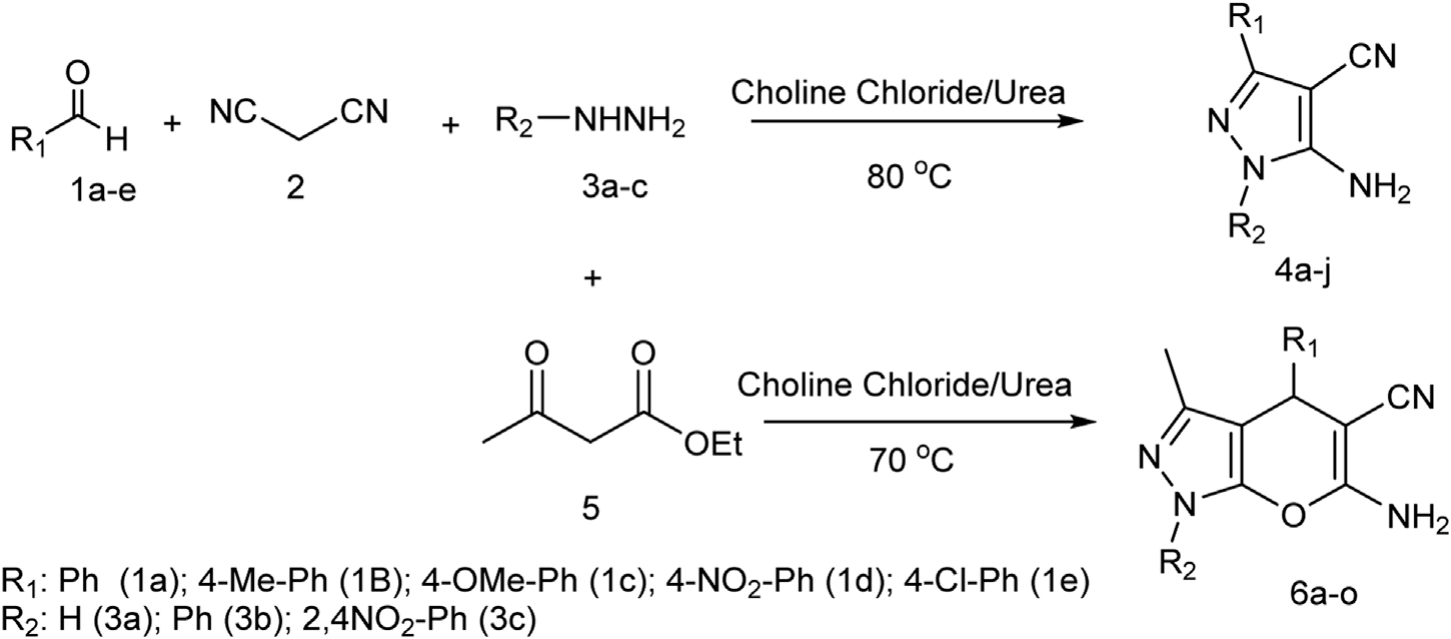
Using choline chloride/urea as green method for synthesis of pyrazole derivatives and pyrano[2,3-c]pyrazole derivatives.

In this study, by using choline chloride/urea as a green and environmentally friendly deep eutectic solvent, in the three-component reaction of malononitrile, aldehyde derivatives, and hydrazine derivatives, novel pyrazole derivatives, and in the four-component reaction of malononitrile, aldehyde derivatives, ethyl acetoacetate, and hydrazine derivatives, novel pyrano[2,3-c]pyrazole derivatives were synthesized (Scheme 1).

In general, the greenness of the reported method, high efficiency, and shorter synthesis time were the advantages of using choline chloride/urea in synthesizing derivatives. Additional information about the synthesis of derivatives is given below.

### 3.1 Three-component synthesis of pyrazole derivatives using choline chloride/urea

The three-component synthesis of pyrazole derivatives used choline chloride/urea, malononitrile, aldehyde derivatives, and hydrazine derivatives were used as raw materials.

For this purpose, according to the method presented in Section 2.2, different ratios of choline chloride and urea were prepared. For the synthesis of 4a, they were tested according to Table 1.

**TABLE 1:** Optimization of molar ratio of choline chloride:urea and reaction temperature in synthesis of pyrazole.

Entry	Product	Molar ratio of choline chloride:urea	Reaction temperature (°C)	Tim (min)	Yield (%)
1	4a	0: 1	80	60	N.R
2	4a	1: 0	80	60	N.R
3	4a	1: 1	80	60	67
4	4a	1: 2	80	20	97
5	4a	1: 3	80	30	82
6	4a	1: 4	80	30	55
7	4a	1: 2	50	30	52
8	4a	1: 2	60	30	76
9	4a	1: 2	70	30	88
10	4a	1: 2	90	30	95
11	4a	1: 2	100	30	90

N.R, no reaction.

In obtaining the optimal conditions (Table 1), in addition to using different ratios of choline chloride and urea, temperatures of 50°C, 60°C, 70°C, 80°C, 90°C, and 100°C degrees Celsius were also tested. It was found that the ratio of 1:2 (choline chloride:urea) at 80°C had the highest efficiency in a shorter time for the synthesis.

Physical parameters such as viscosity (according to ASTM D445 testing method) and melting point for 1:2 choline chloride:urea mixture were obtained according to Table 2.

**TABLE 2:** Physical parameters of a 1:2 ratio of choline chloride:urea deep eutectic solvent.

Viscosity (25°C, cPa)	Melting point temperature (°C)
17.50	20

The above optimal conditions were used for synthesizing other derivatives, according to Table 3. Three of the derivatives reported in Table 3 are novel.

**TABLE 3:** Information on synthesized pyrazole derivatives using choline chloride/urea as green method.

Product code	R_1_	R_2_	Product structure	Reported Mp (°C)	Found Mp (°C)	Synthesis time (min)	Yield (%)
4a	Ph	Ph	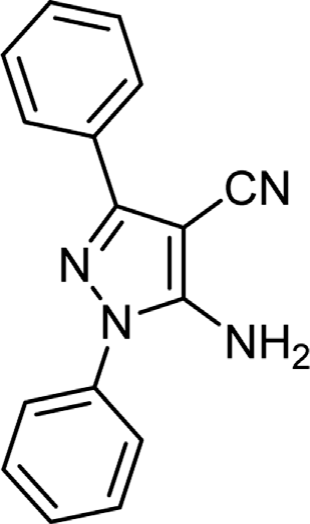	158-159 (Poonam and Singh, 2019)	159-160	20	97
4b	Ph	2,4-NO_2_-Ph	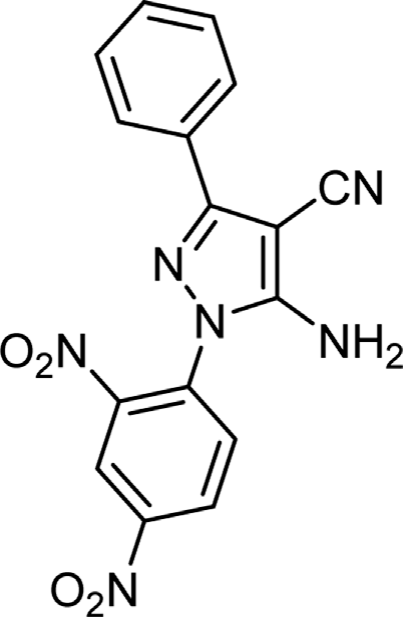	240-142 (Bakhtiarian and Khodaei, 2022)	240-243		95
4c	4-Me-Ph	Ph	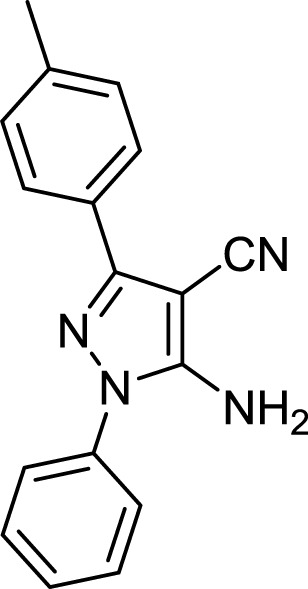	119-121 (Nemati et al., 2021)	120-121		97
4d	4-Me-Ph	2,4-NO_2_-Ph	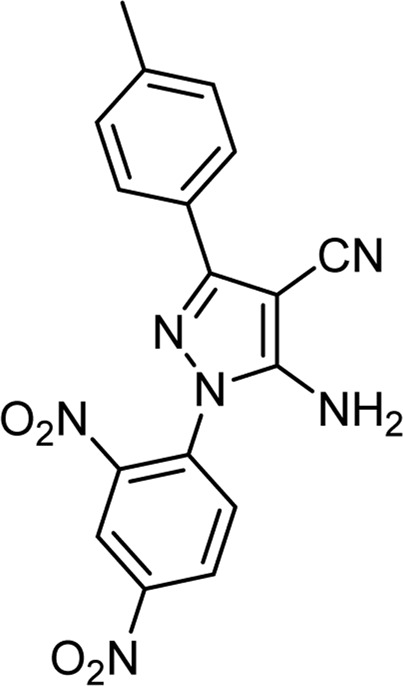	New	245-247		85
4e	4-OMe-Ph	Ph	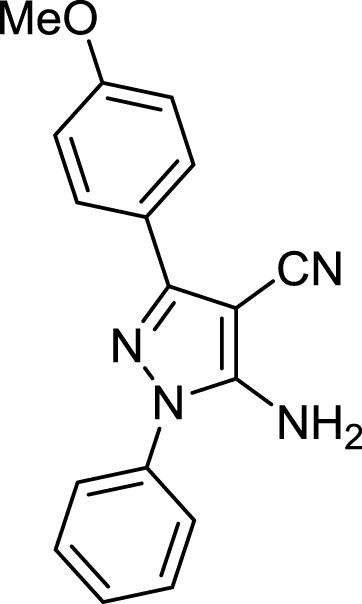	107-109 (Abshirini et al., 2020)	108-110		98
4f	4-OMe-Ph	2,4-NO_2_-Ph	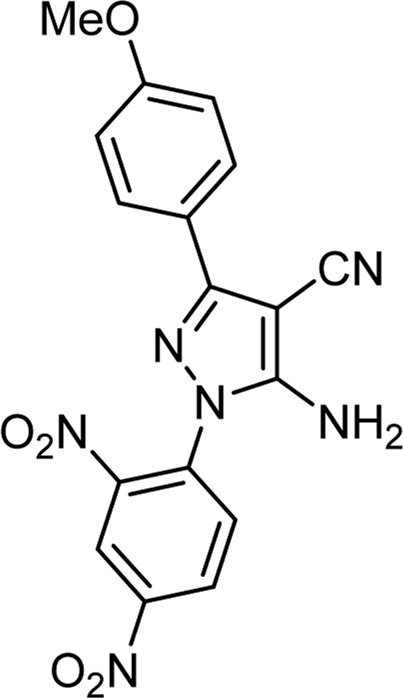	New	264-267		86
4g	4-NO_2_-Ph	Ph	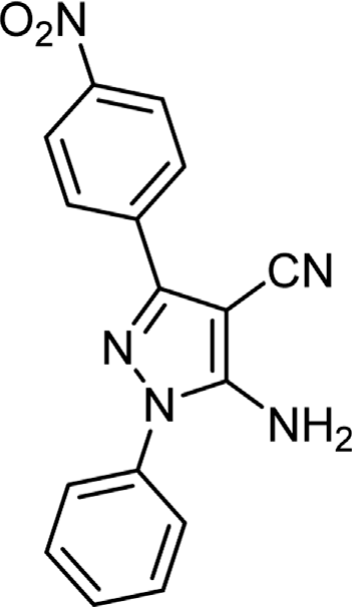	165-168 (Nemati et al., 2021)	166-168		97
4 h	4-NO_2_-Ph	2,4-NO_2_-Ph	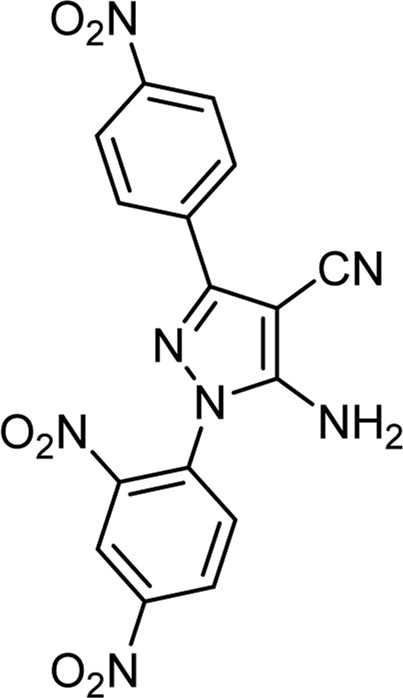	New	266-268		84
4i	4-Cl-Ph	Ph	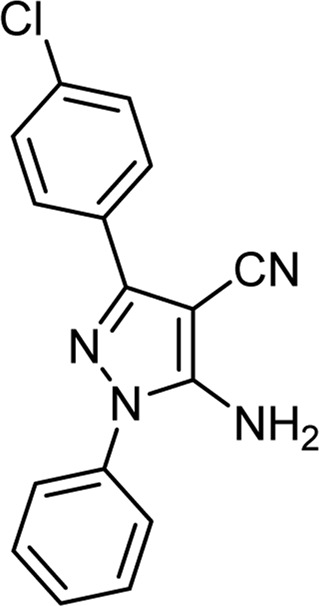	128-130 (Amirnejat et al., 2020)	129-131		98
4j	4-Cl-Ph	2,4-NO_2_-Ph	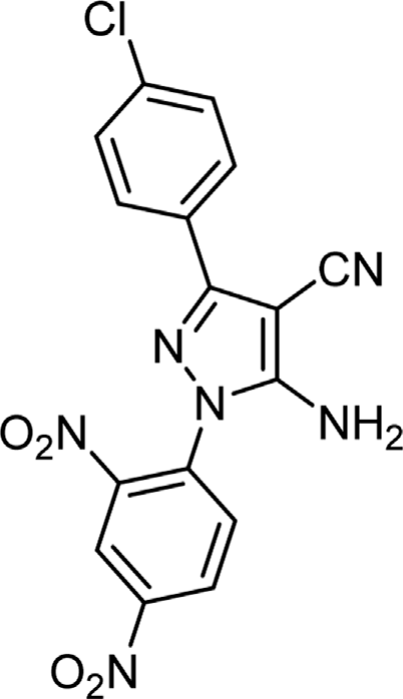	234-236 (Aryan et al., 2017)	235-236		93

Choline chloride:urea (1: 2); 80°C.

The results of ^1^H NMR, ^13^C NMR, and CHNS/O elemental analyzer of the newly synthesized derivatives that confirm their structure are given in the Supplementary Material.

**SCHEME 2:**
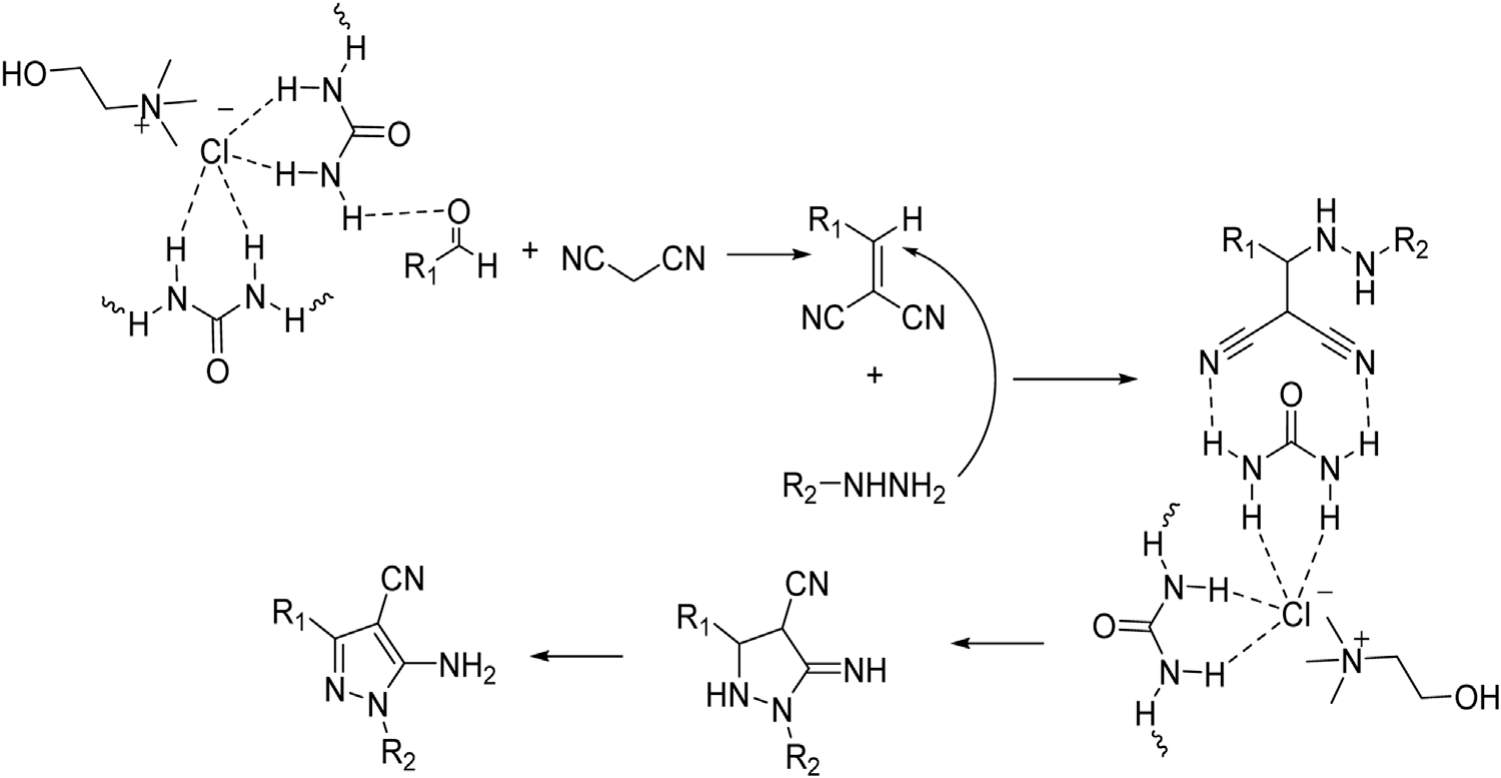
Mechanism for using choline chloride/urea as green method for synthesis of pyrazole derivatives (4a-j).

As the results show, in general, electron-donating substituents attached to hydrazine, such as phenyl, and electron-withdrawing groups attached to the aldehyde, such as nitro, increased the efficiency of the products. Therefore, the following mechanism (Scheme 2) can be proposed for this 3-component reaction, which corresponds to the difference in the results of the product’s efficiency.

So far, many studies have been conducted on the three-component reaction of malononitrile, aldehyde derivatives, and hydrazine derivatives, which led to the synthesis of pyrazole derivatives. The conditions of some recent studies (from 2020 to 2023) compared to the conditions used in this study are given in Table 4.

**TABLE 4:** The previously reported conditions and methods in the synthesis of pyrazole compared to the method presented in this study.

Reported year	Product	Condition (catalyst)	Reaction temperature (°C)	Time	Yield (%)	Reference
2023	4a	Sulfonic acid modified 5-aminotetrazole-1,3,5-triazine-(3-aminopropyl)silylated carbon quantum dots-coated Fe_3_O_4_ magnetic nanoparticles	70	Not reported	95	Ghorbani et al. (2023)
2022	4a	2,6-diamino-1-(4-sulfobutyl)pyridin-1-ium hydrogen sulfate modified pectin nanoparticles	50	30 min	96	Bakhtiarian and Khodaei (2022)
2021	4a	Sulfonic acid-functionalized polyvinyl alcohol	90	3 h	89	Patki et al. (2021)
2020	4a	1,3-disulfoimidazolium trifluoroacetate	80	50 min	94	Abshirini et al. (2020)
This work	4a	Choline chloride:urea	80	20 min	97	-

Ease of reaction conditions and no need for catalyst and solvent, reporting of newly synthesized derivatives, being green, and having high efficiency and shorter synthesis time can be mentioned as advantages of using choline chloride/urea to synthesize derivatives compared to recently reported methods.

### 3.2 Four-component synthesis of pyrano[2,3-c]pyrazole derivatives using choline chloride/urea

In the continuation of this study, choline chloride/urea was also used in the 4-component reaction of malononitrile, aldehyde derivatives, hydrazine derivatives, and ethyl acetoacetate for the synthesis of pyrano[2,3-c]pyrazole derivatives.

The steps for synthesizing derivatives here were the first optimization according to Section 3.1, the results of which are given in Table 5.

**TABLE 5:** Optimization of molar ratio of choline chloride:urea and reaction temperature in synthesis of pyrano[2,3-c] pyrazoles.

Entry	Product	Molar ratio of choline chloride:urea	Reaction temperature (°C)	Tim (min)	Yield (%)
1	6a	0: 1	70	60	N.R
2	6a	1: 0	70	60	N.R
3	6a	1: 1	70	60	42
4	6a	1: 2	70	17	95
5	6a	1: 3	70	30	66
6	6a	1: 4	70	30	54
7	6a	1: 2	50	30	62
8	6a	1: 2	60	30	79
9	6a	1: 2	80	30	90
10	6a	1: 2	90	30	88
11	6a	1: 2	100	30	88

N.R, no reaction.

Here, the optimal conditions for synthesizing pyrano[2,3-c]pyrazole derivatives with high efficiency and shorter time, were the ratio of 1:2 of choline chloride:urea and the reaction temperature of 70°C. With the obtained optimal conditions, other derivatives, including 15 derivatives, of which two were novel, were synthesized (Table 6).

**TABLE 6:** Information on synthesized pyrano[2,3-c]pyrazole derivatives using choline chloride/urea as green method.

Product code	R_1_	R_2_	Product structure	Reported Mp (°C)	Found Mp (°C)	Synthesis time (min)	Yield (%)
6a	Ph	H	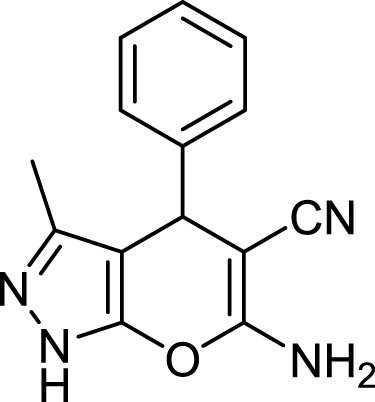	241-243 (Yekke-Ghasemi et al., 2022)	240-242	17	95
6b	Ph	Ph	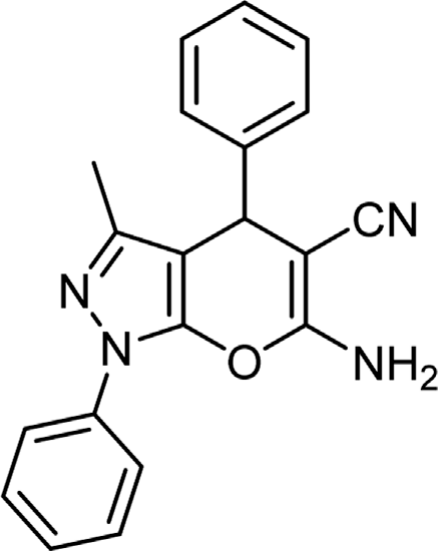	169-171 (Mathavan and Yamajala, 2023)	170-172	15	95
6c	Ph	2,4-NO_2_-Ph	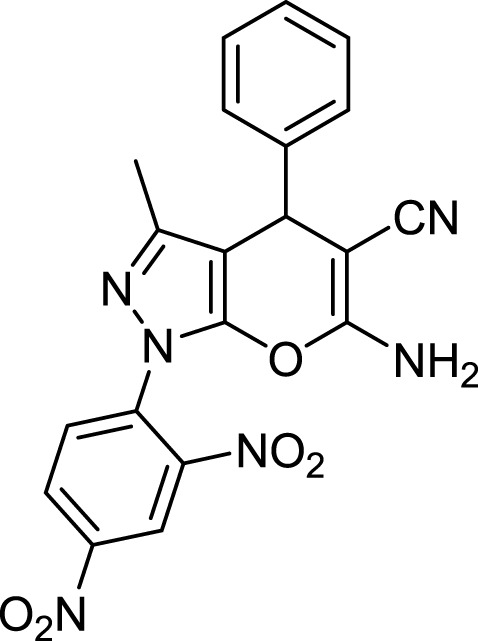	240-243 (Vasava et al., 2019)	241-242	30	90
6d	4-Me-Ph	H	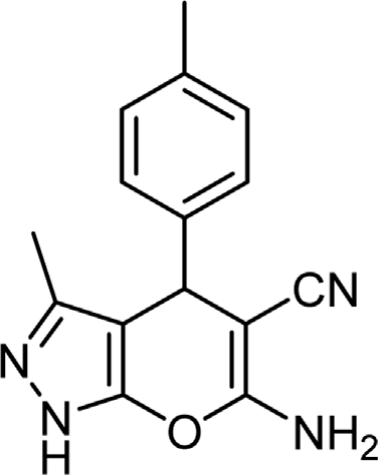	175-177 (El Mejdoubi et al., 2019)	176-178	25	90
6e	4-Me-Ph	Ph	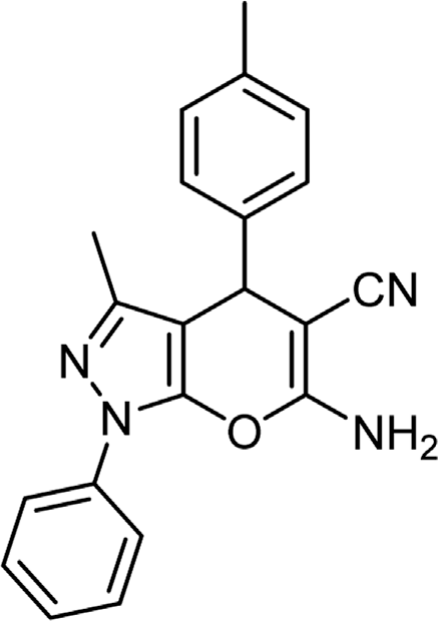	177-179 (Mohamadpour, 2020)	177-180	25	95
6f	4-Me-Ph	2,4-NO_2_-Ph	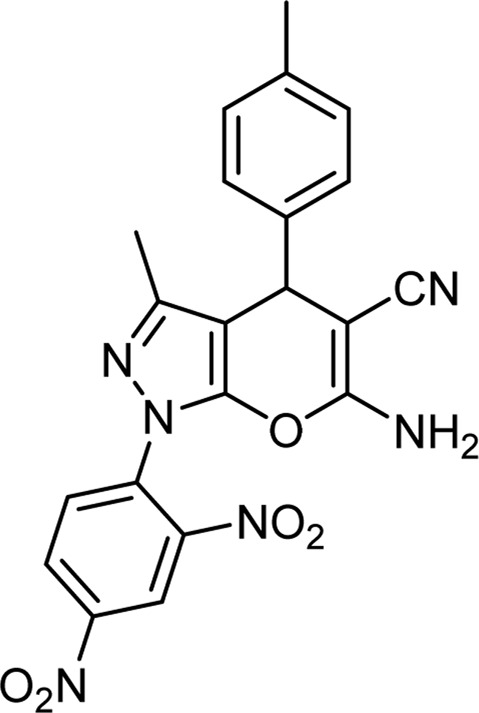	New	270-273	35	85
6g	4-OMe-Ph	H	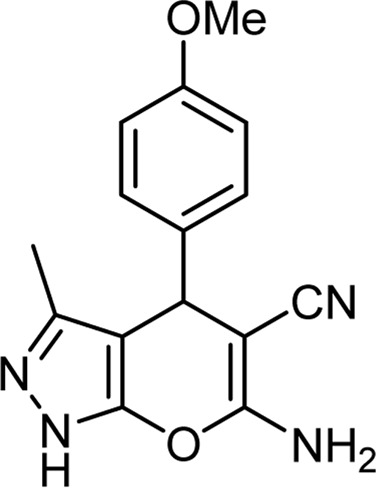	209-211 (Yekke-Ghasemi et al., 2022)	209-212	20	92
6 h	4-OMe-Ph	Ph	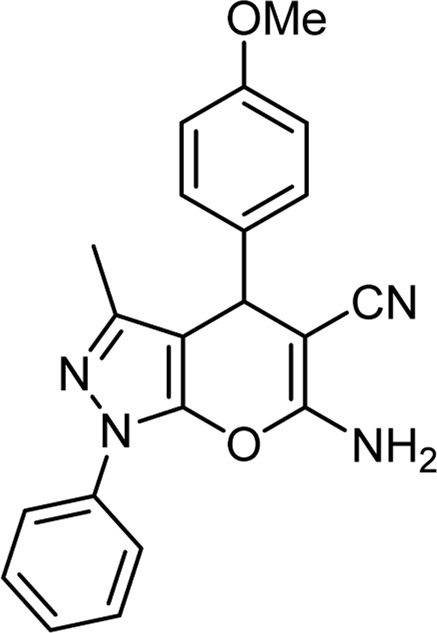	170 (Suryakumari and Sudhakar, 2023)	169-171	18	95
6i	4-OMe-Ph	2,4-NO_2_-Ph	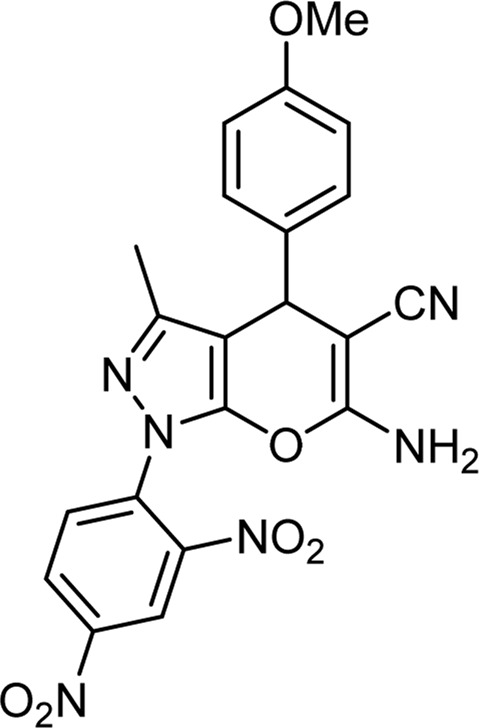	New	288-290	36	85
6j	4-NO_2_-Ph	H	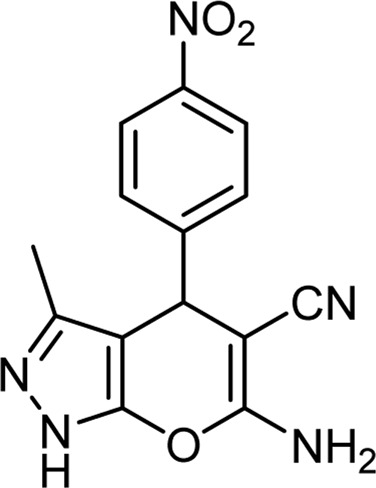	249-251 (Moghaddam et al., 2023)	249-251	20	95
6k	4-NO_2_-Ph	Ph	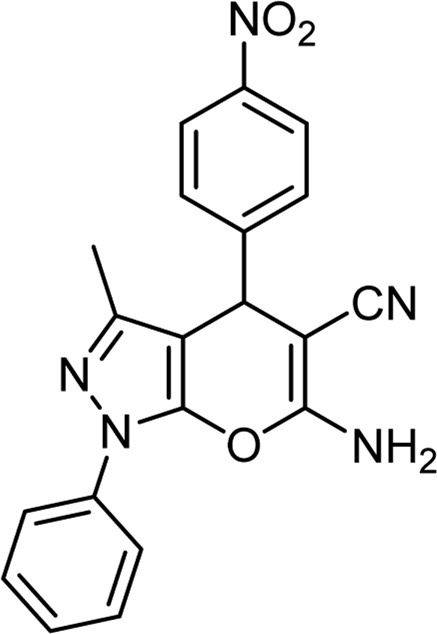	195-197 (Kondabanthini et al., 2022)	194-196	19	95
6L	4-NO_2_-Ph	2,4-NO_2_-Ph	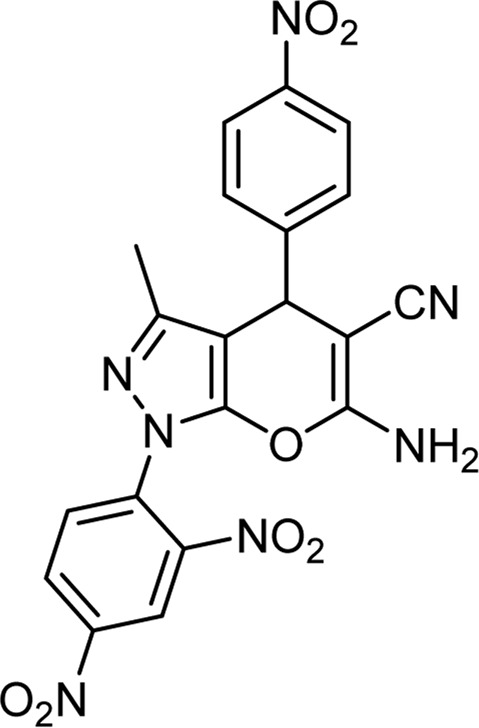	283-285 (Vasava et al., 2019)	284-285	34	88
6m	4-Cl-Ph	H	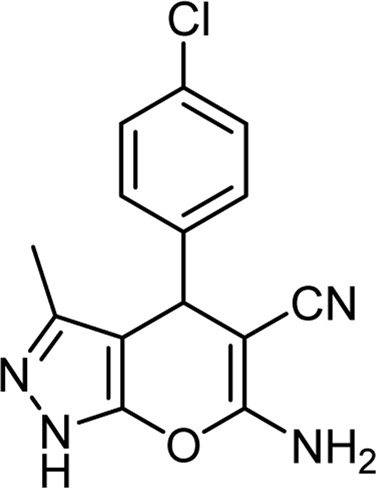	234-236 (Yekke-Ghasemi et al., 2022)	235-236	20	94
6n	4-Cl-Ph	Ph	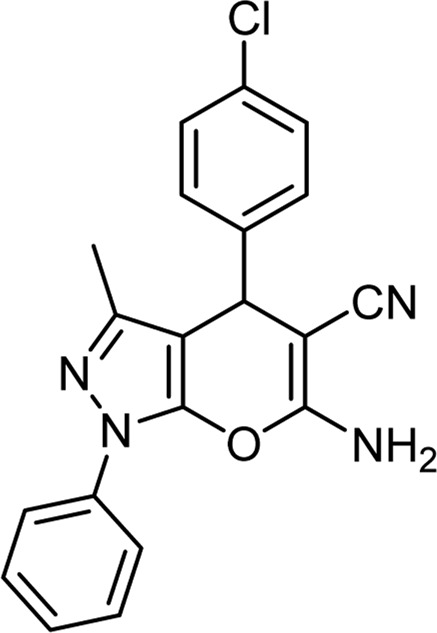	175-177 (Kondabanthini et al., 2022)	175-177	17	97
6o	4-Cl-Ph	2,4-NO_2_-Ph	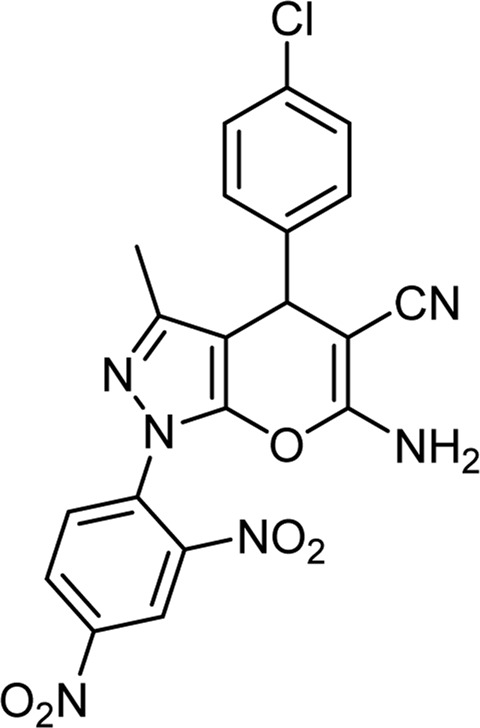	260-265 (Vasava et al., 2019)	263-266	35	80

Choline Chloride: Urea (1: 2); 70°C

The results of ^1^H NMR, ^13^C NMR, and CHNS/O elemental analyzer of the newly synthesized derivatives that confirm their structure are given in the Supplementary Material.

**SCHEME 3:**
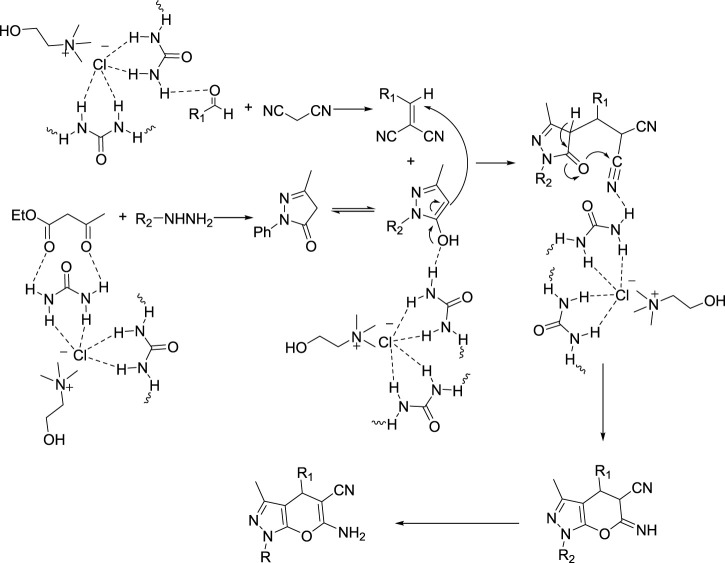
Mechanism for using choline chloride/urea as green method for synthesis of pyrazole pyrano[2,3-c]pyrazole derivatives (6a-o).

Comparing the results of the efficiency with the structure of the raw materials here was also the same as above, and it was observed that electron-donating substituents attached to hydrazine, such as phenyl, and electron-withdrawing groups attached to the aldehyde, such as nitro, increased the efficiency of the products. Therefore, the Scheme 3 mechanism is proposed for this reaction.

The result of the comparison of the method presented in this study to the recent studies in the synthesis of derivatives, as shown in Table 7, is similar to the conclusion for the synthesis of pyrazole derivatives. It can be stated here that ease of reaction conditions, catalyst and solvent not being necessary, newly synthesized derivatives being reported, being green, and having high efficiency and shorter synthesis time can be mentioned as advantages of using choline chloride/urea to synthesize derivatives compared to recently reported methods.

**TABLE 7:** The previously reported conditions and methods in the synthesis of pyrano[2,3-c]pyrazole compared to the method presented in this study.

Reported year	Product	Condition (catalyst)	Reaction temperature (°C)	Time	Yield (%)	Reference
2018	6a	Bael fruit ash	20	15 min	92	Shinde et al. (2018)
2018	6a	Sodium L-ascorbate	Reflux of water	10 min	88	Kiyani and Bamdad (2018)
This work	6a	Choline Chloride: Urea	70	17 min	95	-
2023	6b	Copper(II) oxide incorporated onto montmorilonite-K10 functionalized with tetraethyl orthosilicate, epichlorohydrine, melamine, and 5-sulphosalisilic acid	20	Not reported	90	Karrabi et al. (2023)
2023	6b	Potassium dihydrogenphosphate	100	2 h	95	Mathavan and Yamajala (2023)
2022	6b	Phenylazophenylenediamine-based lanthanum complex supported on silica-coated magnetic nano-Fe_3_O_4_ core-shell nanocatalyst	110	12	90	Ghorbani et al. (2022)
2021	6b	Sugarcane bagasse ash-based silica-supported boric acid	80	35 min	86	Pandey et al. (2021)
2022	6b	Tungstic acid immobilized on zirconium-L-aspartate amino acid metal-organic framework-grafted L-(+)-tartaric acid stabilized magnetic Fe3O4 nanoparticles	60	45 min	92	Khademi et al. (2021)
This work	6b	Choline chloride:urea	70	15 min	95	-

### 3.3 The antimicrobial activity results

In the investigation of antimicrobial and antifungal activity of derivatives, gram-positive, gram-negative, and specialized aquatic strains were used. First, the MIC with a concentration in the range of 1–2048 μg/mL of the derivatives was checked. After obtaining this parameter, its MBC and MFC were acquired. Finally, using the MIC, the IZD parameter of the derivatives on the used strains was obtained.

For further comparison of pyrazole derivatives and pyrano[2,3-c]pyrazole derivatives, the antibacterial properties of gram-positive, gram-negative, and specialized aquatic and fungal strains are shown separately in Tables 8–11.

**TABLE 8:** Antibacterial activity of pyrazole derivatives (4a-j) and pyrazole pyrano[2,3-c]pyrazole derivatives (6a-o) against Gram-positive strains.

Strains		Pyrazole derivatives										Drugs		Pyrano[2,3-c]pyrazole derivatives														
		4a	4b	4c	4d	4e	4f	4g	4h	4i	4j	A	B	6a	6b	6c	6d	6e	6f	6g	6h	6i	6j	6k	6l	6m	6n	6o
10745	(I)	2048	128	-	256	1024	128	512	128	64	64	-	16	512	512	128	1024	256	64	256	128	32	64	64	32	16	16	8
	(II)	4096	256	-	512	2048	128	1024	128	128	128	-	32	1024	512	128	1024	512	128	256	256	64	64	64	64	16	32	16
	(III)	12.6	16.8	-	16.2	12.8	17.3	13.7	15.9	18.2	17.3	-	17.3	14.5	14.9	14.3	15.3	14.8	18.9	17.6	18.5	19.1	18.2	21.2	20.3	22.5	19.9	22.4
29213	(I)	-	-	-	-	-	-	-	-	-	-	16	16	-	-	2048	-	-	512	-	-	256	1024	1024	128	-	-	64
	(II)	-	-	-	-	-	-	-	-	-	-	32	16	-	-	4096	-	-	1024	-	-	512	2048	1024	256	-	-	128
	(III)	-	-	-	-	-	-	-	-	-	-	15.1	16.9	-	-	12.2	-	-	13.8	-	-	13.6	12.3	12.9	14.2	-	-	14.1
19615	(I)	4096	4096	2048	1024	2048	2048	2048	2048	1024	1024	2	8	512	512	16	512	512	8	256	128	4	64	32	4	2	2	2
	(II)	4096	4096	2048	4096	2048	2048	4096	2048	2048	2048	4	16	1024	1024	16	1024	1024	16	512	128	8	128	64	4	4	2	2
	(III)	10.4	10.9	10.7	10.2	10.5	11.3	10.4	10.6	11.8	11.5	23.2	18.4	16.4	18.3	20.8	15.9	19.9	19.7	18.8	19.5	23.1	19.4	21.6	22.5	23.1	22.7	22.9
12386	(I)	-	-	-	-	-	-	-	-	1024	1024	-	4	-	-	64	-	-	32	256	128	16	128	128	8	8	4	2
	(II)	-	-	-	-	-	-	-	-	4096	2048	-	8	-	-	128	-	-	64	512	256	16	128	128	16	8	8	4
	(III)	-	-	-	-	-	-	-	-	18.2	17.5	-	19.1	-	-	21.6	-	-	21.0	17.3	16.6	22.1	16.2	19.4	22.4	21.5	21.7	22.3

(I): Minimum Inhibitory Concentration (μg/mL); (II): Minimum Bactericidal Concentration (μg/mL); (III): Inhibition Zone Diameter (mm);

A, penicillin; B, cefazolin.

**TABLE 9:** Antibacterial activity of pyrazole derivatives (4a-j) and pyrazole pyrano[2,3-c]pyrazole derivatives (6a-o) against gram-negative strains.

Strains		Pyrazole derivatives										Drugs		Pyrano[2,3-c]pyrazole derivatives														
		4a	4b	4c	4d	4e	4f	4g	4h	4i	4j	A	B	6a	6b	6c	6d	6e	6f	6g	6h	6i	6j	6k	6l	6m	6n	6o
9610	(I)	-	-	-	-	-	-	-	-	-	-	-	64	-	-	-	-	-	-	-	-	-	-	-	-	-	-	-
	(II)	-	-	-	-	-	-	-	-	-	-	-	128	-	-	-	-	-	-	-	-	-	-	-	-	-	-	-
	(III)	-	-	-	-	-	-	-	-	-	-	-	12.0	-	-	-	-	-	-	-	-	-	-	-	-	-	-	-
13313	(I)	-	-	-	-	-	-	-	-	1024	1024	-	-	-	-	-	-	-	-	-	-	-	-	-	-	1024	512	512
	(II)	-	-	-	-	-	-	-	-	4096	2048	-	-	-	-	-	-	-	-	-	-	-	-	-	-	1024	1024	512
	(III)	-	-	-	-	-	-	-	-	10.7	12.5	-	-	-	-	-	-	-	-	-	-	-	-	-	-	13.6	12.4	12.3
15442	(I)	-	-	-	-	-	-	-	-	-	-	-	-	-	-	2048	-	-	2048	4096	2048	1024	1024	1024	512	512	256	128
	(II)	-	-	-	-	-	-	-	-	-	-	-	-	-	-	4096	-	-	2048	4096	4096	1024	2048	2048	1024	512	256	256
	(III)	-	-	-	-	-	-	-	-	-	-	-	-	-	-	11.8	-	-	11.2	10.1	11.4	12.3	11.9	11.5	12.4	13.6	13.8	13.5
19606	(I)	-	-	-	-	-	-	-	-	-	1024	-	-	-	-	-	-	-	-	-	-	-	-	-	-	-	-	256
	(II)	-	-	-	-	-	-	-	-	-	2048	-	-	-	-	-	-	-	-	-	-	-	-	-	-	-	-	512
	(III)	-	-	-	-	-	-	-	-	-	11.5	-	-	-	-	-	-	-	-	-	-	-	-	-	-	-	-	12.3

(I): Minimum Inhibitory Concentration (μg/mL); (II): Minimum Bactericidal Concentration (μg/mL); (III): Inhibition Zone Diameter (mm);

A, penicillin; B, cefazolin.

**TABLE 10:** Antibacterial activity of pyrazole derivatives (4a-j) and pyrazole pyrano[2,3-c]pyrazole derivatives (6a-o) against specialized aquatic strains.

Strains			Pyrazole derivatives										Drugs		Pyrano[2,3-c]pyrazole derivatives														
			4a	4b	4c	4d	4e	4f	4g	4h	4i	4j	A	B	6a	6b	6c	6d	6e	6f	6g	6h	6i	6j	6k	6l	6m	6n	6o
Gram- positive	29178	(I)	2048	256	1024	512	1024	512	1024	128	32	32	32	16	4096	-	512	2048	-	256	2048	1024	512	1024	512	128	32	32	32
		(II)	4096	512	2048	1024	2048	512	1024	256	64	64	64	64	4096	-	1024	4096	-	512	4096	2048	512	1024	1024	256	64	64	64
		(III)	11.7	13.8	12.5	11.9	13.0	13.4	11.8	14.2	15.9	16.7	16.6	14.2	10.5	-	1024	11.2	-	13.8	12.1	15.1	14.2	14.4	15.7	13.6	15.7	15.3	15.9
	43921	(I)	-	512	-	1024	2048	512	512	256	256	128	2	-	-	-	128	2048	-	64	512	256	32	256	256	16	4	2	2
		(II)	-	512	-	2048	4096	1024	1024	256	512	256	4	-	-	-	256	4096	-	128	1024	512	128	256	256	32	16	4	2
		(III)	-	12.9	-	11.5	11.9	13.6	14.2	15.4	16.5	17.7	19.7	-	-	-	21.8	10.2	-	20.7	17.6	19.3	21.5	19.8	17.5	22.9	22.6	23.5	23.1
Gram- negative	29473	(I)	-	-	-	-	-	-	-	-	2048	2048	-	32	-	-	1024	-	-	1024	4096	4096	1024	4096	2048	512	256	64	64
		(II)	-	-	-	-	-	-	-	-	4096	4096	-	64	-	-	1024	-	-	2048	4096	4096	1024	4096	4096	512	512	256	128
		(III)	-	-	-	-	-	-	-	-	11.6	11.1	-	15.9	-	-	12.8	-	-	11.3	12.2	10.3	12.6	10.7	10.9	12.4	13.7	14.5	15.1
	15947	(I)	-	-	-	-	-	-	-	-	-	-	8	-	-	-	256	-	-	128	2048	1024	64	512	512	16	16	8	4
		(II)	-	-	-	-	-	-	-	-	-	-	16	-	-	-	512	-	-	128	2048	2048	128	1024	1024	64	16	16	8
		(III)	-	-	-	-	-	-	-	-	-	-	17.5	-	-	-	17.3	-	-	16.4	15.5	14.9	18.7	17.7	16.2	17.8	19.1	19.5	19.6

(I): Minimum Inhibitory Concentration (μg/mL); (II): Minimum Bactericidal Concentration (μg/mL); (III): Inhibition Zone Diameter (mm);

A, penicillin; B, cefazolin.

**TABLE 11:** Antifungal activity of pyrazole derivatives (4a-j) and pyrazole pyrano[2,3-c]pyrazole derivatives (6a-o).

Strains		Pyrazole derivatives										Drugs		Pyrano[2,3-c]pyrazole derivatives														
		4a	4b	4c	4d	4e	4f	4g	4h	4i	4j	A	B	6a	6b	6c	6d	6e	6f	6g	6h	6i	6j	6k	6l	6m	6n	6o
10231	(I)	-	1024	-	4096	2048	1024	2048	1024	512	512	-	32	-	4096	512	-	1024	512	2048	1024	512	1024	512	256	128	128	64
	(II)	-	1024	-	4096	4096	2048	2048	2048	1024	1024	-	64	-	4096	1024	-	2048	1024	2048	2048	512	2048	1024	512	256	256	128
	(III)	-	12.6	-	11.4	11.7	12.1	11.8	13.0	15.9	15.5	-	21.6	-	11.7	16.6	-	12.3	16.9	13.5	14.1	16.3	15.9	16.9	17.8	17.6	17.3	18.4
32045	(I)	-	-	-	-	-	-	-	-	-	-	-	64	-	-	-	-	-	-	-	-	-	-	-	-	-	-	-
	(II)	-	-	-	-	-	-	-	-	-	-	-	128	-	-	-	-	-	-	-	-	-	-	-	-	-	-	-
	(III)	-	-	-	-	-	-	-	-	-	-	-	17.2	-	-	-	-	-	-	-	-	-	-	-	-	-	-	-
7601	(I)	-	-	-	-	-	-	-	-	512	256	-	64	-	-	-	-	-	-	-	-	-	-	-	-	256	256	128
	(II)	-	-	-	-	-	-	-	-	1024	512	-	64	-	-	-	-	-	-	-	-	-	-	-	-	512	256	256
	(III)	-	-	-	-	-	-	-	-	12.7	12.8	-	20.8	-	-	-	-	-	-	-	-	-	-	-	-	13.0	14.6	14.1
1022	(I)	-	512	4096	2048	2048	1024	1024	512	256	256	-	32	2048	-	128	2048	-	64	1024	512	64	512	256	64	16	16	16
	(II)	-	1024	4096	4096	2048	1024	2048	1024	512	256	-	64	4096	-	256	4096	-	128	1024	1024	128	512	512	128	32	32	32
	(III)	-	12.2	14.6	14.8	13.1	13.7	13.8	12.8	15.9	15.5	-	20.3	10.8	-	17.9	11.8	-	18.6	1024	16.3	17.4	17.7	1024	18.2	19.5	19.8	19.3

(I): Minimum Inhibitory Concentration (μg/mL); (II): Minimum Fungicidal Concentration (μg/mL); (III): Inhibition Zone Diameter (mm);

A, tolnaftate; B, terbinafine.

Table 8 shows the results of investigating the antibacterial properties of the derivatives on the evaluated gram-positive strains.

Table 9 shows the results of investigating the antibacterial properties of the derivatives on the evaluated gram-negative strains.

Table 10 shows the results of investigating the antibacterial properties of the derivatives on the evaluated specialized aquatic strains.

Table 11 shows the results of investigating the antibacterial properties of the derivatives on the evaluated fungal strains.

The highest effectiveness in antibacterial activity between pyrazole derivatives and pyrano[2,3-c]pyrazole derivatives was related to 6o.

**FIGURE 1:**
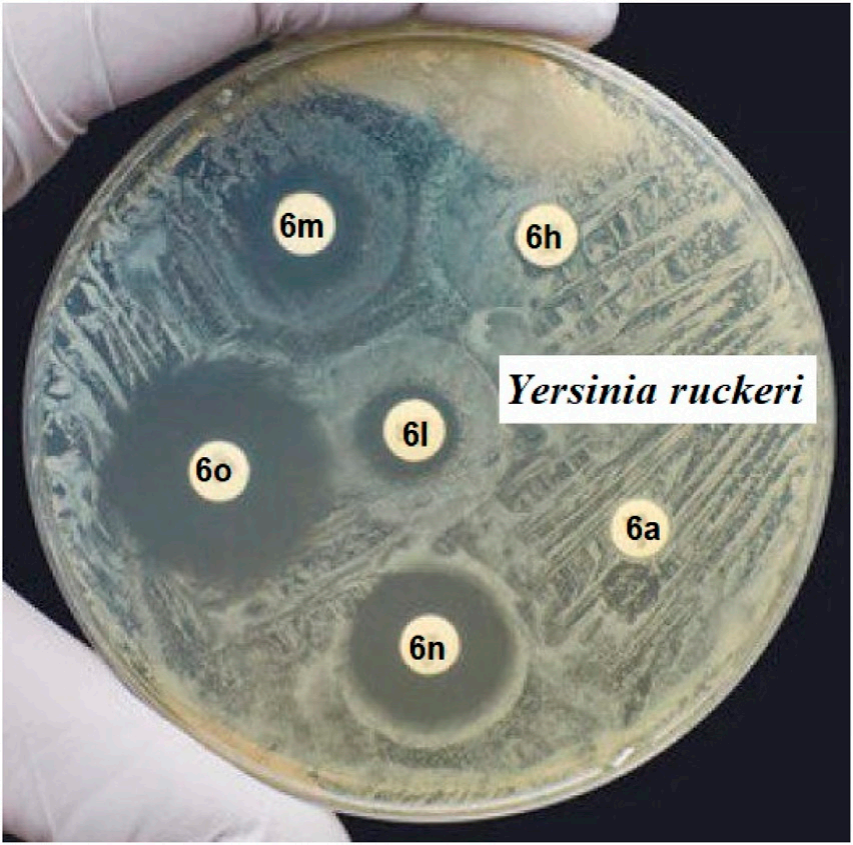
The IZD value results of some pyrano[2,3-c]pyrazole derivatives against *Yersinia ruckeri*.

The IZD value results of some pyrano[2,3-c]pyrazole derivatives compared to *Yersinia ruckeri* are given in Figure 1.

In general, the order of effectiveness of pyrazole derivatives was as follows:

4j > 4i > 4h > 4f > 4d≈4b > 4g > 4e > 4b≈4a

In general, the order of effectiveness of pyrano[2,3-c]pyrazole derivatives was as follows:

6o > 6n≈6m > 6l > 6i > 6f≈6c > 6j≈6k > 6h≈6g > 6e≈6d≈6b≈6a

By comparing the results of Tables 8–11, the pyrano[2,3-c]pyrazole derivatives, in general, are more effective than pyrazole derivatives because pyrano[2,3-c] pyrazoles contain two heterocyclic rings (pyran and pyrazole). Another general conclusion that can be drawn is that the order of effectiveness of derivatives is as described for compounds containing chlorine, nitrogen, and methoxy. These results can be justified based on previous studies on the effectiveness and antimicrobial properties of chlorine and nitrogen. The important point in determining antimicrobial activities is to compare them with commercial drugs, such as penicillin, cefazolin, Tolnaftate, and Terbinafine. As the results of Tables 8–11 show, some derivatives, such as 6o in many studied strains, are much more effective than tested drugs, which shows the value of synthetic compounds.

## 4 Conclusion

Choline chloride/urea was used as a deep eutectic solvent in the one-pot method and during the multicomponent reaction in synthesizing pyrazole derivatives and pyrano[2,3-c]pyrazole derivatives. The greenness of the synthesis method, the synthesis of new derivatives, and the characteristics of high efficiency and less time were among the advantages and novel findings of this study compared to previously reported methods. The effectiveness on gram-positive, gram-negative, and specialized aquatic strains and fungal species is one of the novel findings of this project. In the investigation of antimicrobial activity, the highest effectiveness was observed in derivative 6o, which contained chlorine and nitrogen, where MIC and MBC values between 2 and 512 μg/mL were observed. In addition, 6o showed higher effectiveness in some strains than drugs in the market, such as penicillin, cefazolin, Tolnaftate, and Terbinafine. Finally, the relationship between the antimicrobial properties of the derivatives and their structure were observed and reported.

## Data Availability

The original contributions presented in the study are included in the article/Supplementary material, further inquiries can be directed to the corresponding author.
